# Clinical education stressors in medical trainees in Shahid Sadoughi University of Medical Sciences, Yazd

**Published:** 2016-01

**Authors:** MAHDIEH MOMAYYEZI, HOSSEIN FALLAHZADEH, MOHAMMAD MOMAYYEZI

**Affiliations:** 1 Research center of prevention and epidemiology of non-communicable disease, Public Health School, Shahid Sadoughi University of Medical Sciences, Yazd, Iran;; 2 School of Medicine, Shahid Sadoughi University of Medical Sciences, Yazd, Iran

**Keywords:** Clinical, Education, Stressors, Medical trainees

## Abstract

**Introduction:**

Stress is an important factor in the educational process. Teaching and learning are stressful processes. This stress can affect one's ability and change his/her performance. The purpose of this study was to investigate stressors of clinical education from the perspective of medical students in Yazd University of Medical Sciences.

**Methods:**

This descriptive-analytic study was conducted in Yazd University of Medical Science during year 2014-2015. The sample size was 170 medical students who were selected randomly. The data were collected by a questionnaire including four components: interpersonal relationship, educational environment, clinical experience and the unpleasant emotions. A significance level of 0.05 was considered for analysis. The statistical analyses included descriptive statistics, ANOVA and T-tests, using SPSS software, version 14.

**Results:**

The results showed that the highest domain score belonged to interpersonal relationship (3.33±0.3) followed by unpleasant emotions domain (3.3±0.3). The lowest domain score of clinical education stressors was educational environment (3.12±0.1). The results showed that the mean score of interpersonal relationship domain was more in women than in men (p<0.05).

**Conclusion:**

The relationship between teachers and students is an effective factor in all dimensions of clinical education stressors. So proper measures such as the promotion of scientific awareness of teachers and educational staff about *factors* that lead to *stress and the best way to communicate with students* should be taken to reduce the students’ stress.

## Introduction

Stress is an integral part of human life (1). It can lead to mental and physical illness (2). Stress is defined as the subjective perception and interpretation of a situation that is beyond one’s ability and can cause health problems (3).

Stress is an important factor in the educational process. Teaching and learning are stressful processes. The results of several studies suggest that medical students face a lot of stressors during college life. In addition to theoretical education stressors, medical students are exposed to stressors of clinical education and hospital environments. The hospital is one of the most stressful workplaces because hospital personnel are involved in the life and death of human beings (4).

Clinical education is considered as facilitating activities in the clinical environment and its purpose is to create measurable changes in the students during clinical care. Stress in clinical education can have adverse effects such as academic underachievement and inappropriate behaviors in students (5). This stress can affect one's ability and change his/her performance. An inverse relationship was observed between stress and academic performance (6). In addition to these issues, students may display inappropriate behavior when facing stress, such as smoking, suicide attempts and use of alcohol, and drugs abuse. Akbari et al. in their study indicated that 52% of dental students had abnormal stress from mild to very severe (7).

Several studies have shown a variety of reasons for stress in the clinical environment. Nazari et al. found that the main source of stress in nursing students was warning to the students by teachers in the presence of personnel and physicians (5). Abazari et al. in their study indicated that the difference between theoretical education and clinical education was the major cause of stress (8). Some researchers have tried to identify stressors in clinical education through qualitative studies. They concluded that the main cause of tension in students was interpersonal communication between teachers and students (9). Also, other studies showed that the principal sources of stress in the clinical care include: lack of knowledge and skill, and insufficient familiarity with the hospital processes (7).

So, the educational managers must provide a stress-free environment for learning through awareness of the sources of stress and then limit them. In this connection the purpose of the present study was to investigate stressors of clinical education from the perspective of medical students in Yazd University of Medical Sciences. 

## Methods

This descriptive-analytic study was conducted in Yazd University of Medical Science during year 2014-2015. The sample size was determined to be 170 medical students, who were selected randomly. The inclusion criteria for the students included passing at least one clinical course in hospital and satisfaction to participate in this study. The data were collected by a questionnaire, which was designed based on scientific articles (10).

The first section of the questionnaire included demographic variables (age, sex). The second part of the questionnaire included 40 questions that evaluated the clinical education stressors, categorized into four components: Interpersonal relationship (14 items), educational environment (6 items), clinical experience (11 items) and the unpleasant emotions (9 items). A 5-point Likert-type scale was used to measure all questions: 1= never, 2= very low, 3= low, 4= high, 5= very high. Researchers used the Cronbach's alpha to determine the reliability of the questionnaire. The face validity was examined using a panel of experts. Cronbach's alpha was calculated for the whole questionnaire and then for all dimensions. Cronbach's alpha for the whole questionnaire was appropriate (α=0.97). Also, Cronbach's alpha for all dimensions of questionnaire was acceptable. 

The construct validity was determined, using the factor analysis method. The results of the factor analysis, dimensions of the questionnaire and questions for each dimension are shown in table 1.

Initially, the researchers explained the purposes of this study to the students, and then the questionnaires were completed by the participants. The completed questionnaires were collected anonymously. 

The statistical analysis was performed, using SPSS software, version 14. The statistical analysis included descriptive statistics (mean, standard deviation), ANOVA and t-tests. 

**Table1:** Factor analysis for clinical education stressors questionnaire by using principal components

**Row**	**Dimensions**	**Factor** **loading**	**Eigen values**	**% of variance**
**Factor 1** ** (** **unpleasant emotions** **)**			11.51	19.19
**1**	Lack of interest in primary care practice	0.80		
**2**	Conflict with the patient and their families	0.78		
**3**	Lack of interest in clinical activities	0.78		
**4**	Lack of confidence	0.74		
**5**	Fear of teacher's assessment	0.70		
**6**	Fear of mental harm to the patients	0.68		
**7**	Fear of the transmission of infectious diseases from patients to students	0.67		
**8**	Fear of physical harm to the patients	0.66		
**9**	Fear of lack of knowledge and skills	0.44		
**Factor 2 (educational environment** **)**			9.31	15.52
**10**	Lack of appropriate places for study and conferences	0.87		
**11**	The variety of disease cases for learning in hospital	0.75		
**12**	Various teachers in theoretical and clinical sectors	0.66		
**13**	The presence of students in different courses by the patient bed	0.65		
**14**	Not clear goals and tasks in the clinical environment	0.54		
**15**	The large number of students in every part of the hospital	0.45		
**Factor ** **3 (Interpersonal relationship)**			9.18	15.3
**16**	Interaction and behavior between students and teachers	0.78		
**17**	Interaction and behavior between students and Physicians	0.77		
**18**	Interaction and behavior between students and patients	0.71		
**19**	Multiple demands of the patients’ families from students	0.63		
**20**	Multiple demands of patients from students	0.62		
**21**	Teachers’ reminders to students in the presence of personnel and physicians	0.60		
**22**	Calling out the students loudly in hospital	0.60		
**23**	Teachers’ reminders to students in the presence of other students	0.59		
**24**	Teachers’ reminders to students in the presence of patients and their families	0.55		
**25**	Medical staff’s reminders to teachers about lack of cooperation by students	0.55		
**26**	Presence of teachers in any part of the hospital and monitoring students’ performance	0.54		
**27**	Various questions from students by patients	0.53		
**28**	Interaction and behavior between students and medical staff	0.52		
**29**	Lack of cooperation between medical staff and students	0.48		
**Factor ** **4 (clinical** ** experience)**			5.47	9.13
**30**	Prescription of injection drugs	0.79		
**31**	Prescription of oral drugs	0.73		
**32**	Inadequate care and treatment by doctors	0.68		
**33**	Inadequate care and treatment by medical staff	0.66		
**34**	Forcing students to do things outside of their duties	0.56		
**35**	Sense of teachers’ responsibility for patient's treatment	0.51		
**36**	Multiple tasks delegated to students	0.49		
**37**	No use of theoretical knowledge in the clinical environment	0.48		
**38**	Observation of suffering of patients with poor prognosis	0.47		
**39**	Teachers’ Lack of knowledge and skills	0.43		
**40**	Students’ sense of responsibility for patients’ treatment	0.34		

## Results

The results showed that the mean age was 21.73±0.79 years; also, 57.1 % of the students were female. The highest domain score belonged to interpersonal relationship (3.33±0.3), followed by unpleasant emotions domain (3.3±0.3). The lowest domain score of clinical education stressors was for the educational environment (3.12±0.1). Table 2 shows the mean and standard deviation for each of the dimensions of clinical education stressors.

**Table2:** Mean of dimensions of clinical education stressors

**Dimensions of life style**	**Mean±SD**
Interpersonal relationship	3.33±0.3
Educational environment	3.25±0.5
Clinical experience	3.12±0.1
Unpleasant emotions	3.30±0.3

According to the results, the most important stressor in clinical training in the interpersonal communication domain was "interaction and behavior between students and teachers". The most important stressors in educational environment domain were '' Not clear goals and tasks in the clinical environment'' followed by "the large number of students in every part of the hospital". The most important stressors in clinical experience domain were "students’ sense of responsibility for patient's treatment ", followed by '' Observation of suffering of patients with poor prognosis ''. The most important stressors in unpleasant emotions domain were '' Fear of physical harm to the patients '', followed by "Fear of lack of knowledge and skills in clinical care ".

Comparing male and female students, we found that the highest domain score in female students belonged to the interpersonal relationship (3.63±0.9) and the lowest domain of clinical education stressors in them was clinical experience (3.17±0.7). Also, the educational environment domain (3.07±0.07) was the highest stressful domain in male students and interpersonal relationship domain (2.93±0.2) was the lowest stressful domain in them. 

The t-test analysis showed that the mean score of all stressful domains were higher in women than in men, but this relationship was only significant in the interpersonal relationship domain. The mean of stressors domains according to gender is shown in table 3.

**Table3:** The mean of stressors domains according to gender

**Dimensions of life style**	**Female**	**Male**	**p**
Interpersonal relationship	3.63±0.9	2.93±0.2	0.004
Educational environment	3.39±0.5	3.07±0.07	0.24
Clinical experience	3.17±0.7	3.04±0.1	0.60
Unpleasant emotions	3.45±0.8	3.09±0.01	0.24

Significant level: 0.05

## Discussion

The results showed that the highest domain score of stressor factors was interpersonal relationship, and "interaction and behavior between students and teachers" was the most important stressor in this domain. This part included the relationship between students, teachers, patients and hospital staff. Investigation of the stressor factors in this domain showed that communication between students and teachers was the most stressful factor. This finding was consistent with other studies (11). Also, Timmins and Kaliszer showed that inappropriate relationship between teachers and students was one of the factors affecting stress among students (12). Shahini et al. found that communication skills were one of the important tasks of teachers and an integral part of clinical education (13). Tang et al. pointed out that the important features for an effective teacher is confidence and respect to students (14). Also, other studies in this field have shown that disrespect of teachers to the students in the presence of others was an important factor causing stress (15). The large number of students, lack of physical space, and too many tasks of teachers were the causes of this problem. Appropriate behavior with students is very important to increase their interest in a clinical training environment.

Also, the findings showed that the mean score of this domain was higher in women. Taebi et al. found that stress in female students were more than in male students in communicating with teachers (11). So, some activities to reduce the stress should be done (such as the promotion of scientific awareness of teachers and educational staff about factors that lead to stress, and the best way for communicating with students).

The results showed that the second domain score of stressor factors was unpleasant emotions, and ''fear of physical harm to the patients'' and "fear of lack of knowledge and skills" were the most important stressors in this domain. Kermansaravi et al. showed that this domain was the highest domain between clinical education stressors (16). Kleehammer et al. found that students worried about the mistakes they made due to insufficient knowledge and skills (17). Moridi et al. showed that insufficient skill for taking care of patients was one of the main stressful resources (10). Melo et al. showed that lack of skills was the most important stressor in clinical courses (18). So a suitable environment for students to transform the theory to practice in clinical training without stress should be provided.

The third domain score of stressor factors was educational environment and ''not clear goals and tasks in the clinical environment and the presence of uncertainty'' and then "the large number of students in every part of a hospital" were the most important stressors in this domain. Shafie et al. found that teachers should create a proper understanding of the tasks for the students through proper planning and provide correct information for the students and support them emotionally (19). Also, poor attention to the students’ duties is an important source of stress in students (5). Parvan et al. showed that there was a negative significant correlation between students' stress and nurse in clinical setting educators’ effectiveness. They suggested that all nursing members should be provided with a suitable educational plan to resolve educational problems in clinical settings (20).

Educational planners should help to accelerate the learning process of students by determining a proper time schedule. On the other hand, a balance between the number of students at the university and educational facilities is a major step forward to solve the problem.

The fourth domain score of stressor factors was clinical experience, and "sense of responsibility of student for patients’ treatment" and ''observation of suffering of patients with poor prognosis'' were the most important stressors in this domain. Moridi et al. showed that the students’ sense of responsibility for patient's treatment was the main source of stress in clinical experience domain in students of nursing; also observation of suffering of patients with poor prognosis '' was the most important stressor in this domain in students of operating room (10). Other studies suggested that lack of clinical expertise was one of the major causes of stress (15).

Limitation of the study was lack of control on the stressor events that may happen in the person's life during the present study. 

## Conclusion

According to the results of this study, there were several stressors in medical students who were studying in the clinical environment. The most important of these factors were interpersonal relationship and unpleasant emotions. Based on the findings of this study, it is necessary to resolve the problems by providing a suitable environment in teaching hospitals, and clarifying the duties of students at the beginning of the learning process, determining teachers' expectations from the students, and holding stress management workshops according to the needs of the students. 
